# A Driving and Control Scheme of High Power Piezoelectric Systems over a Wide Operating Range

**DOI:** 10.3390/s20164401

**Published:** 2020-08-07

**Authors:** Tianyue Yang, Yuanfei Zhu, Zhiwei Fang, Haoyu Wu, Wanlu Jiang, Ming Yang

**Affiliations:** 1 Department of Instrument Science and Engineering, Shanghai Jiao Tong University, Shanghai 200240, China; thomas-yang@sjtu.edu.cn (T.Y.); zhuyuanfei@sjtu.edu.cn (Y.Z.); sjtufzw@sjtu.edu.cn (Z.F.); wuhaoyu@alumni.sjtu.edu.cn (H.W.); 2 School of Mechanical Engineering, Yanshan University, Qinhuangdao 066000, China; wljiang@ysu.edu.cn

**Keywords:** high-power piezoelectric systems, parallel resonance tracking, vibration amplitude control, transformer ratio arm bridge, pulse-based phase detector

## Abstract

Significant variation in impedance under a wide range of loads increases the difficulty of frequency tracking and vibration control in high-power piezoelectric systems (HPPSs). This paper proposed a wide operating range driving and control scheme for HPPSs. We systematically analyzed the impedance characteristics and deduced the load optimization frequency. In order to provide sufficient drive capability, the inverter combined with an LC matching circuit is configured. With the aid of a transformer ratio arm bridge (TRAB) combined with a proposed pulse-based phase detector (PBPD), the proposed scheme can control the vibration amplitude and keep parallel resonance status under a wide range of loads. Experiments conducted under actual operating conditions verify the feasibility of the proposed scheme under the modal resistance range from 7.40 to 500 Ω and the vibration range from 20% to 100%. Moreover, with the aid of a laser displacement sensor, our scheme is verified to have a vibration amplitude control accuracy better than 2% over a tenfold load variation. This research could be helpful for the driving and control of HPPSs operating in a wide range.

## 1. Introduction

High-power piezoelectric systems (HPPSs) are used in a large range of applications, such as ultrasonic welding, cutting and actuators [1,2,3,4]. In high-power situations, it is crucial to effectively convert the electrical energy into the mechanical vibration in the piezoelectric transducers (PTs) [5,6,7,8]. Generally, researchers excite PTs at their mechanical resonance frequency, that is, the series resonance frequency ($f_{s}$), where the minimum excitation voltage is required [1,9]. However, as the vibration amplitude increases, an additional loss near $f_{s}$ degrades the performance of the PTs in high power [10,11]. It is attributed to the dielectric loss which is related to the input current in HPPSs [12]. Therefore, exciting the PTs at the parallel resonance frequency ($f_{p}$) with the minimum excitation current can achieve the optimal efficiency. For the HPPSs operating under high vibration amplitude, high excitation voltage is required at $f_{p}$ [12]. However, different materials and processes result in wide range of loads in some ultrasonic machining, such as welding and cutting [13,14]. The load increases the required excitation voltage, leading to the risk of electrical breakdown. Therefore, avoiding the excessive rise up of excitation voltage under high load conditions becomes a challenge for HPPSs. Meanwhile, the wide range of loads has a significant impact on the impedance characteristics, leading to another challenge in providing sufficient drive capability over the operating range.

In most ultrasonic systems, piezoelectric transducers (PTs) need to be excited in the resonant mode [1,9]. Besides, different vibration amplitudes are required for different processing materials [14,15]. For the PT working at light loading conditions, the excitation voltage and current are almost in phase at $f_{s}$ and $f_{p}$, while the impedance reaches the minimum and maximum near $f_{s}$ and $f_{p}$, respectively [16]. Therefore, the working frequency can be tracked by a phase locking loop (PLL) or impedance extremum search [17]. Meanwhile, the vibration amplitude can be controlled through current and voltage driving modes at $f_{s}$ and $f_{p}$, respectively [9,16]. However, as the load increases, two pairs of zero phase frequencies and the impedance extreme frequencies gradually deviate from $f_{s}$ and $f_{p}$ [17]. In order to enlarge the operating load range, schemes based on the impedance or admittance calculation are proposed, such as the maximum target impedance scheme and the admittance circle tracking scheme [1,18]. However, these schemes require complicated software operations to calculate the frequency deviation and the vibration amplitude. With the aid of a transformer ratio arm bridge (TRAB), a vibration amplitude signal is obtained online in an ultrasonic motor control scheme under different operating conditions [19]. Moreover, this signal is in phase with the excitation current at $f_{p}$, so the parallel resonance detection can be achieved by detecting the phase between the vibration and the excitation current signals. However, the excitation current can be extremely discontinuous and harmonic-rich in the HPPSs over a wide operating range, leading to a new challenge for phase detection.

In order to drive the HPPSs at load optimization frequency under a wide range of loads with controllable vibration amplitudes, a driving and control scheme is proposed in this paper. We first analyze the impact of different loads on the impedance characteristics, and propose the load optimization frequency tracking mode. Second, in order to provide the sufficient drive capability, the effect of the inverter combined with an LC matching circuit is analyzed under different operating conditions. Then, the pulse-based phase detector (PBPD) is proposed to overcome the challenge of phase detection over a wide operating range. Finally, the proposed scheme is verified under actual operating conditions in terms of the frequency tracking and vibration control.

## 2. Electrical Architecture

### 2.1. Equivalent Models

**Figure 1:**
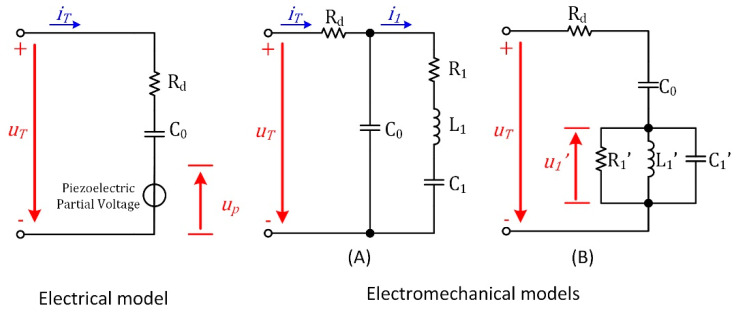
Electrical and Electromechanical models of high-power piezoelectric systems (HPPSs).

An HPPS can be characterized by electromechanical models (A and B) deduced from an electrical model, as shown in Figure 1 [12].

In the electromechanical models, the dielectric property is characterized by $C_{0}$ and $R_{d}$. For model A, which is similar to the classic Butterworth-Van Dyke (BVD) model [20,21], the series $R_{1}$, $L_{1}$ and $C_{1}$ characterize the modal damping, mass and stiffness, respectively. Under the steady state of sinusoidal excitation, the model A can be converted to the model B [19]. In this model, the parameters can be calculated by

(1) $$\{\begin{array}{c} R_{1}^{\prime }=\frac{1}{B_{0}^{2}R_{1}}\\ L_{1}^{\prime }=\frac{C_{p}}{B_{0}^{2}}\\ C_{1}^{\prime }=B_{0}^{2}L_{1} \end{array},$$

where

(2) $$\{\begin{array}{c} B_{0}=\omega C_{0}\\ C_{p}=\frac{C_{0}C_{1}}{C_{0}+C_{1}} \end{array}.$$

In this paper, some electrical characteristics under sinusoidal excitation can be expressed in complex vectors form written in bold letters, such as

(3) $$\{\begin{array}{c} U_{1}^{\prime }=U_{1}^{\prime }e^{j\omega t}\\ U_{T}=U_{T}e^{j\omega t}\\ I_{T}=I_{T}e^{j\omega t} \end{array},$$

where $U_{1}^{\prime }$ is the partial voltage of the parallel RLC part, and $U_{T}$ and $I_{T}$ are the excitation voltage and current of the transducer, respectively. Here, $Z_{1}^{\prime }=U_{1}^{\prime }/I_{T}$ is defined as the impedance of the parallel RLC part, which satisfies the relationship

(4) $$Z_{1}^{\prime }=1/\left(\frac{1}{R_{1}^{\prime }}+j\omega L_{1}^{\prime }+\frac{1}{j\omega C_{1}^{\prime }}\right).$$

Then, we define θ to be the phase angle of $Z_{1}^{\prime }$, that is, the phase between $U_{1}^{\prime }$ and $I_{T}$. Therefore, it can be inferred that θ=0 at $f_{p\;}$ without the influence of $R_{1},$ because the parallel RLC part resonates at $f_{p}$ according to

(5) $$f_{p}=\frac{1}{2\pi \sqrt{L_{1}^{\prime }C_{1}^{\prime }}}.$$

Moreover, when comparing with the electrical model, it can be noted that the partial voltage $u_{1}^{\prime }$ in the model B corresponds to the piezoelectric voltage $u_{p}$ in the electrical model, which is proportional to the vibration amplitude [12]. Therefore, $u_{1}^{\prime }$ can be used for vibration and parallel resonance detection without additional calculation.

In this paper, we use a DUKANE 20 kHz 3300 W piezoelectric transducer, a 1:1.5 transducer amplitude transformer, and a φ70 mm plastic welding horn to construct a typical HPPS used for ultrasonic welding. The parameters of the HPPS are shown in Table 1. It should be noted that, in actual operating conditions, $R_{1}$ increases from 7.40 Ω under no load condition to 200~500 Ω under high load conditions.

### 2.2. Impedance Characteristics Analysis

To analyze the influence of wide range of loads in an HPPS, the variations in $U_{T}$ and $I_{T}$ are calculated under a constant vibration. First, we set $U_{1}^{\prime }$ to be a typical value 1700 V, and $I_{T}$ can be calculated as

(6) $$I_{T}=\left|\frac{U_{1}^{\prime }}{Z_{1}^{\prime }}\right|.$$

Then, $U_{T}$ is deduced to be

(7) $$U_{T}=\left|I_{T}\left(Z_{1}^{\prime }+R_{d}+1/j\omega C_{0}\right)\right|.$$

**Figure 2:**
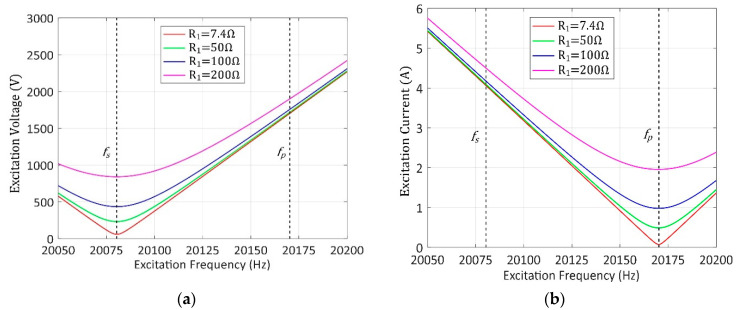
Variations in excitation voltage (**a**) and current (**b**) under the same vibration amplitude.

Therefore, the variations in $U_{T}$ and $I_{T}$ under different excitation frequencies are calculated and demonstrated in Figure 2a,b, respectively, in which $R_{1}$ is taken as four different typical values of 7.4, 50, 100 and 200 Ω. This analysis shows that $I_{T}$ increases linearly while $U_{T}$ increases slightly at $f_{p}$ as $R_{1}$ increases. Therefore, it can be inferred that the wide range of loads leads to an equal variation range of $I_{T}$ at $f_{p}$. Furthermore, the current variation range is further expanded to over a hundred times when the vibration amplitude is controlled from 20% to 100% in our system.

**Figure 3:**
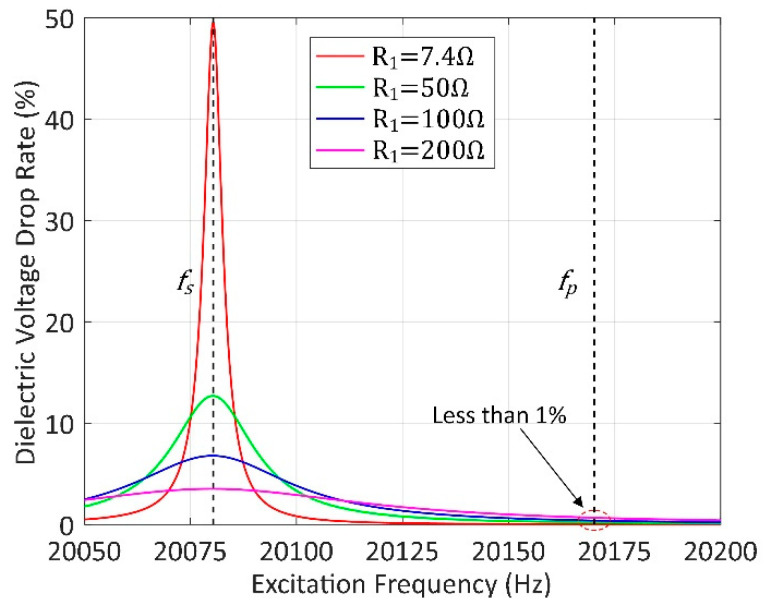
Dielectric voltage drop rate under different frequencies and loads.

Moreover, we analyze the influence of $R_{d}$ from the perspective of the dielectric voltage drop rate calculated as $I_{T}R_{d}/U_{T}$, as shown in Figure 3. It shows that $R_{d}$ has the greatest impact at $f_{s}$, especially under light load conditions. However, the dielectric voltage drop rate decreases to less than 1% near $f_{p}$. Therefore, the influence of $R_{d}$ on the impedance characteristics can be ignored in our scheme.

**Figure 4:**
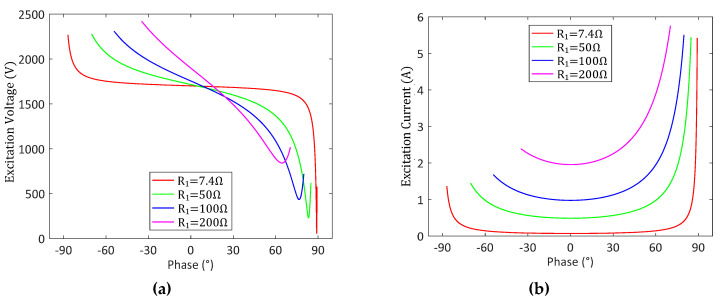
Variations in excitation voltage (**a**) and current (**b**) under different phase θ.

Further, we analyze the variations of $U_{T}$ and $I_{T}$ near $f_{p}$ from the perspective of the phase θ under the constant vibration amplitude ($U_{1}^{\prime }$ equals to 1700 V), as shown in Figure 4a,b, respectively. The relationships of $U_{T}$ and $I_{T}$ are deduced as

(8) $$I_{T}=\frac{U_{1}^{\prime }}{R_{1}^{\prime }\cos\theta },$$

(9) $$U_{T}=\left|U_{1}^{\prime }+I_{T}\left(R_{d}+1/j\omega C_{0}\right)\right|.$$

The curves of $U_{T}$ shows that $U_{T}$ increases with $R_{1}$ at $f_{p}$, and decreases with a slope positively related to $R_{1}$. as θ increases. Meanwhile, it shows that $I_{T}$ is very close to the minimum near the zero phase due to the inverse relationship with cosθ. The analysis above suggests that slight phase difference has little influence on the impedance characteristics. Especially, an appropriate phase difference, such as 20°, can prevent the rise of $U_{T}$ under high load conditions with a negligible rise of $I_{T}$. Therefore, we suggest the load optimization frequency to be slightly lower than $f_{p}$ with θ near 20° in our typical HPPS.

## 3. Proposed Driving Scheme

### 3.1. Electrical Architecture

**Figure 5:**
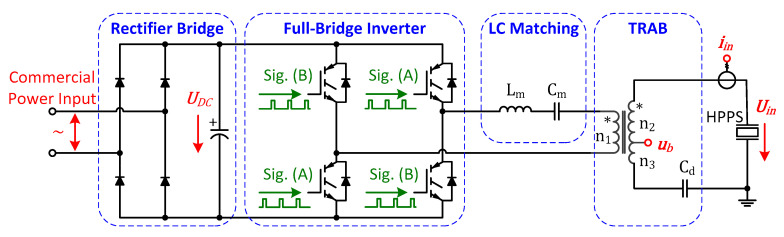
Electrical architecture of the proposed scheme.

The proposed scheme contains a rectifier bridge, a full-bridge inverter, an LC matching circuit and a transformer, as shown in Figure 5.

The commercial power (220 V 50 Hz) is rectified into the DC power $U_{DC}$, and then inverted to the AC power in ultrasonic frequency. A series LC matching circuit is used for DC isolating and harmonic filtering. More importantly, the specific configuration of $L_{m}$ and $C_{m}$ is also related to impedance matching and vibration excitation, which are analyzed in the next sector. Since a transformer ratio arm bridge (TRAB) is easy to be intergraded with little impact on the electrical circuit [16], it is adopted to detect the partial voltage $u_{1}^{\prime }$ online. A tap is drawn from the secondary side of the transformer with the coil turns satisfying $n_{2}\gg n_{3}$, and a detection capacitor $C_{d}$ is connected into the circuit, which satisfies

(10) $$C_{d}=\frac{n_{2}+n_{3}}{n_{3}}C_{0}.$$

Therefore, the bridge voltage, that is, the transformer tap voltage $u_{b}$ satisfies the relationship

(11) $$U_{b}=\frac{n_{3}}{n_{2}+n_{3}}U_{T}-j\omega C_{d}I_{T}=\frac{n_{3}}{n_{2}+n_{3}}U_{1}^{\prime }.$$

In the proposed scheme, we configure $n_{1}=22$, $n_{2}=155$, $n_{3}=4$ and $C_{d}=752\;\mathrm{nF}$ according to the analysis above.

### 3.2. Electrical Properties

**Figure 6:**
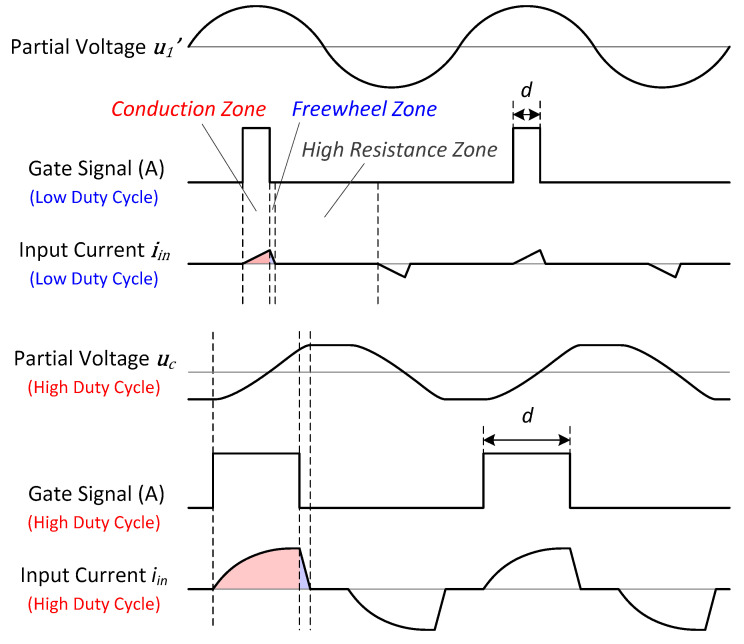
Typical waveforms of the proposed scheme.

Under the driving of the gate signals, the inverter continuously changes the switching state, as shown in Figure 6. The input current to the HPPS $i_{in}$ rises up in the conduction zones and falls down in the freewheeling zones, then $i_{in}$ becomes zero and the inverter enters high resistance zones. As the duty cycle d of the gate signals increases, the conduction zones become wider, leading to the increase in the vibration amplitude. However, the situation is much different under different d.

**Figure 7:**
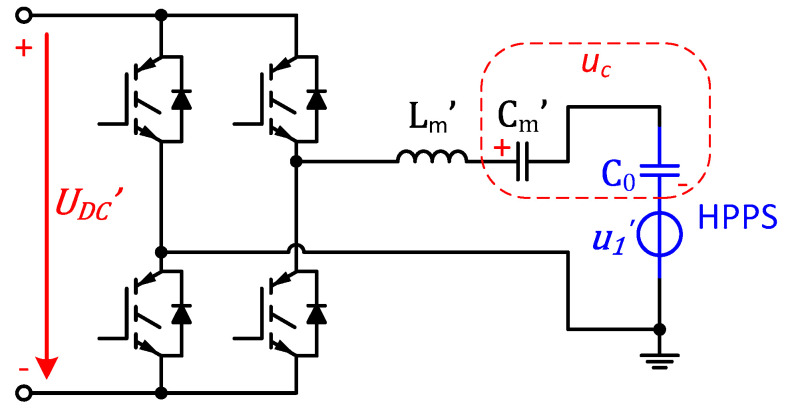
Equivalent electrical circuit of the proposed scheme.

In order to analyze the electrical properties under the steady state near $f_{p}$, the electric architecture is simplified by equivalent transformation, as shown in Figure 7, leading to

(12) $$\{\begin{array}{c} L_{m}^{\prime }=k^{2}L_{m}\\ C_{m}^{\prime }=\frac{1}{k^{2}}C_{m}\\ U_{DC}^{\prime }=kU_{DC} \end{array},$$

where k is the transformer ratio. $L_{m}^{\prime }$, $C_{m}^{\prime }$ and $U_{DC}^{\prime }$ are the matching inductance, matching capacitance and DC voltage after transformation, respectively. We also define $u_{C}$ as the sum voltage of $C_{m}^{\prime }$ and $C_{0}$.

First, we analyze the situations with low duty cycle d. Under these conditions, $i_{in}$ is always small, leading to negligible $u_{C}$. Therefore, in the conduction zones, there exists

(13) $$\frac{di_{in}}{dt}=\frac{1}{L_{m}^{\prime }}\left(U_{DC}^{\prime }-u_{1}^{\prime }\right),$$

while there exists

(14) $$\frac{di_{in}}{dt}=\frac{1}{L_{m}^{\prime }}\left(-U_{DC}^{\prime }-u_{1}^{\prime }\right)$$

in the freewheel zones. $i_{in}$ and other related waveforms are shown in Figure 6. Since only the fundamental wave can excite the modal vibration, we use the Fourier series to extract it in $i_{in}$ to consider as the excitation current $i_{T}$ of the HPPS, which can be calculated as

(15) $$i_{T}=\frac{\omega_{p}}{\pi }\int_{-\frac{\pi }{\omega_{p}}}^{\frac{\pi }{\omega_{p}}}i_{in}\cos\left(\omega_{p}t\right)dt\cos\left(\omega_{p}t\right)+\frac{\omega_{p}}{\pi }\int_{-\frac{\pi }{\omega_{p}}}^{\frac{\pi }{\omega_{p}}}i_{in}\sin\left(\omega_{p}t\right)dt\sin\left(\omega_{p}t\right).$$

When we define $u_{1}^{\prime }$ to be in phase with $\sin\left(\omega_{p}t\right)$, and $i_{in}$ should also be in phase. Therefore, the first term in Equation (15) equals to zero. On the other hand, the conduction and freewheeling zones are concentrated near the peak of $\sin\left(\omega_{p}t\right)$. Therefore, Equation (15) can be approximated as

(16) $$i_{T}=\frac{\omega_{p}}{\pi }\int_{-\frac{\pi }{\omega_{p}}}^{\frac{\pi }{\omega_{p}}}\left|i_{in}\right|dt\sin\left(\omega_{p}t\right),$$

By consideration of Equations (13), (14) and (16), it can be deduced that

(17) $$i_{T}=4\left(A+B\right)\sin\left(\omega_{p}t\right),$$

where A and B correspond to the effects of the conduction zones and the freewheeling zones, respectively, deduced as

(18) $$\{\begin{array}{c} A=\frac{d^{2}}{2L_{m}^{\prime }f_{p}}\left(U_{DC}^{\prime }-U_{1}^{\prime }\right)\\ B=\frac{d^{2}}{2L_{m}^{\prime }f_{p}}\frac{U_{DC}^{\prime }-U_{1}^{\prime }}{U_{DC}^{\prime }+U_{1}^{\prime }}\left(U_{DC}^{\prime }-U_{1}^{\prime }\right) \end{array}.$$

According to $U_{1}^{\prime }=I_{T}R_{1}^{\prime }$ at $f_{p}$, the relationship between $U_{1}^{\prime }$ and d can be derived as

(19) $$U_{1}^{\prime }=\frac{-\left(1+2D\right)+\sqrt{1+12D+4D^{2}}}{2}U_{DC}^{\prime },$$

where

(20) $$D=\frac{d^{2}R_{1}^{\prime }}{2L_{m}^{\prime }f_{p}}.$$

Therefore, $L_{m}$ is inversely proportional to $U_{1}^{\prime }$ under the same load and duty cycle.

**Figure 8:**
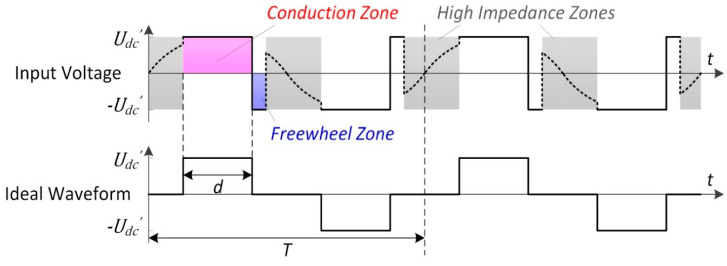
Waveforms of the input voltage and the corresponding bidirectional pulse under high duty cycle.

For the conditions where the inverter is driven with high duty cycle d, the capacitor voltage $u_{C}$ is considerable, leading to Equations (21) and (22) in the conduction zones and freewheel zones, respectively. This mechanism leads to the heaping of the current waveform, as shown in Figure 6, which greatly increases the excitation current to the HPPS. Although it is difficult to analyze the current waveform further, the behavior of the inverter can be analyzed from the perspective of the input voltage $u_{in}$ under these situations. The conduction zones gradually dominate in the waveform of $u_{in}$, approaching to a bidirectional pulse wave with increasing d, as shown in Figure 8.

(21) $$\frac{di_{in}}{dt}=\frac{1}{L_{m}}\left(U_{DC}^{\prime }-u_{C}-u_{1}^{\prime }\right).$$

(22) $$\frac{di_{in}}{dt}=\frac{1}{L_{m}}\left(-U_{DC}^{\prime }-u_{C}-u_{1}^{\prime }\right).$$

Here, it is important to configure LC matching circuit to offset the capacitive reactance of $C_{0}$ and make the circuit purely resistive at $f_{p\;}$, satisfying

(23) $$2\pi f_{p\;}L_{m}-\frac{1}{2\pi f_{p\;}C_{m}}=k^{2}\frac{1}{2\pi f_{p\;}C_{0}}.$$

Therefore, it can be noted that the partial voltage $u_{1}^{\prime }$ is equal to fundamental wave of the output voltage of the inverter under the matching condition above, satisfying

(24) $$U_{1}^{\prime }=\frac{4U_{DC}^{\prime }}{\pi }\sin\pi d.$$

This relationship suggests that the scheme can excite $U_{1}^{\prime }$ to the maximum value of $\frac{4}{\pi }kU_{DC}$ without being affected by the load.

**Figure 9:**
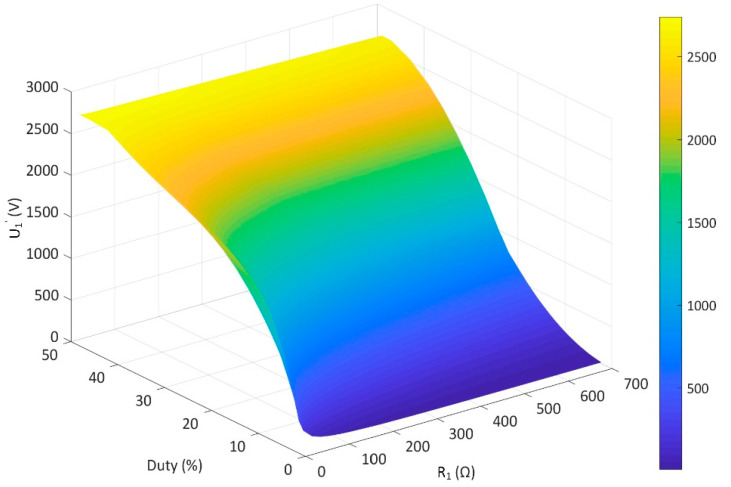
Simulation result of vibration amplitude under different loads and duty cycle.

Due to the analyzed above, we configure $L_{m}=143\;\mu H$ and $C_{m}=870\;\mathrm{nF}$. Here, we use MATLAB/Simulink (MathWorks, Natick, MA, USA, 2017b) to simulate the relationship among d, $R_{1}$ and $U_{1}^{\prime }$, as shown in Figure 9. It shows that $U_{1}^{\prime }$ increases smoothly with d under different loads. This result verifies the drive capability of our scheme under a wide range of loads with adjustable vibration amplitude.

## 4. Detection and Control

### 4.1. Pulse Based Phase Detector

In order to maintain constant vibration amplitude and keep operating near $f_{p}$, the amplitude of $u_{1}^{\prime }$ and its phase θ with $I_{in}$ need to be detected. Owing to the TRAB integrated in our scheme, $u_{1}^{\prime }$ is extracted via the signal $u_{b}$, which is strong and pure under most conditions. Therefore, it can be reliably digitized through the zero-crossing comparator after squelch. Meanwhile, $I_{in}$ is detected by a feed-through current transformer. However, filtering and digitizing $i_{in}$ is difficult due to the discontinuous and harmonic-rich in the large variation range.

**Figure 10:**
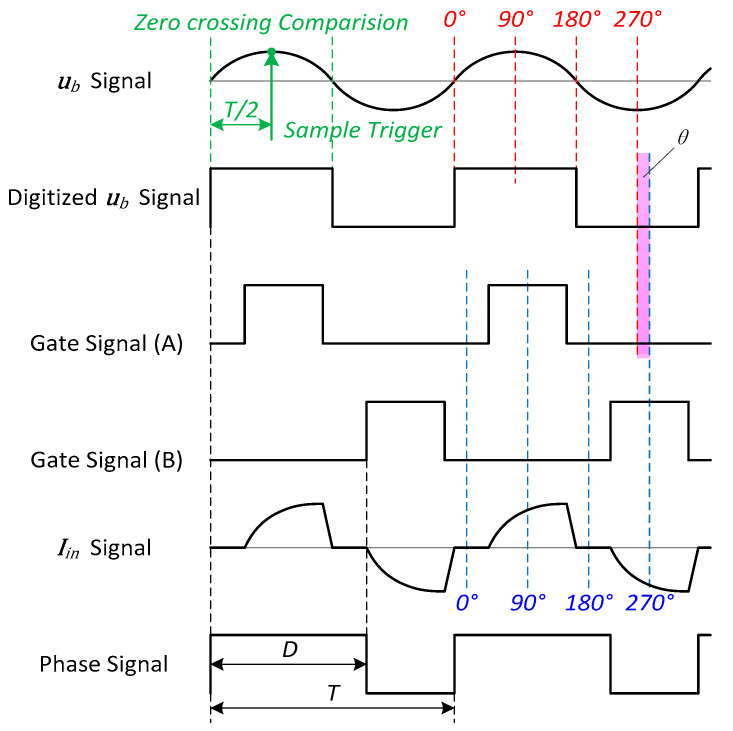
Timing diagram of the relevant signals of pulse-based phase detector (PBPD).

**Figure 11:**
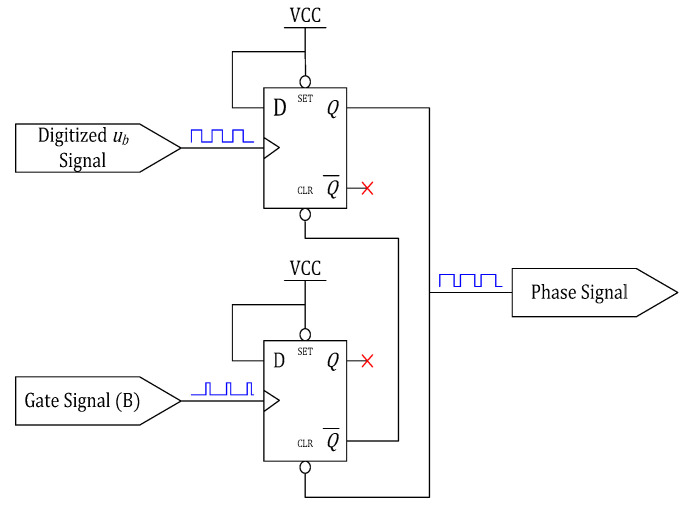
Structure of the D flip-flops-based detector.

In this scheme, a pulse-based phase detector (PBPD) is proposed. Since the input current $i_{in}$ almost occurs in the conduction zones, a PWM gate signal is used instead of the $i_{in}$. A classical digital phase detector based on D flip-flops is adopted to generate a phase detection signal. The timing diagram of the relevant signals and the phase detector circuit are shown in Figure 10 and Figure 11, respectively.

In the proposed scheme, we use the gate signal B but not A to avoid the potential competitive risk, and define the pulse center to be 270°. Therefore, the phase θ can be calculated though the relationship

(25) $$\theta =360{}^{\circ}*\frac{D+d/2}{T}-270{}^{\circ},$$

where D and T are the pulse width and the cycle of the phase signal, respectively. Affected by the variation in the current waveform under different conditions, the phase θ obtained by PBPD has a small deviation, which will be further analyzed in the experiment.

This signal is also used in the detection of $U_{b}$, which triggers a T/4 peak sampling timer on the rising edge, as shown in Figure 10.

### 4.2. Control Realization

Due to the perturbation of $U_{DC}$ and variation of load, a vibration close loop is needed in the proposed scheme. As d is deduced to be in positive correlation to $U_{1}^{\prime }$, the control logic is established as

(26) $$\Delta d=K_{p,d}\left(U_{b,target}-U_{b}\right),$$

where $U_{b}$ is the feedback parameter, $U_{b,target}$ is the vibration target voltage, and $K_{p,d}$ is the proportional control parameter in the vibration amplitude controller.

Moreover, due to the change of temperature and the coupling stiffness caused by the loads, a frequency closed loop needs to be executed in parallel with the vibration close loop. Since θ is inversely related to frequency and equal to zero at $f_{p\;}$, the control logic is established as

(27) $$\Delta f=K_{p,f}\theta ,$$

where $K_{p,f}$ is the proportional control parameter in the frequency controller.

## 5. Experimental Results

### 5.1. Frequency Tracking Verification

**Figure 12:**
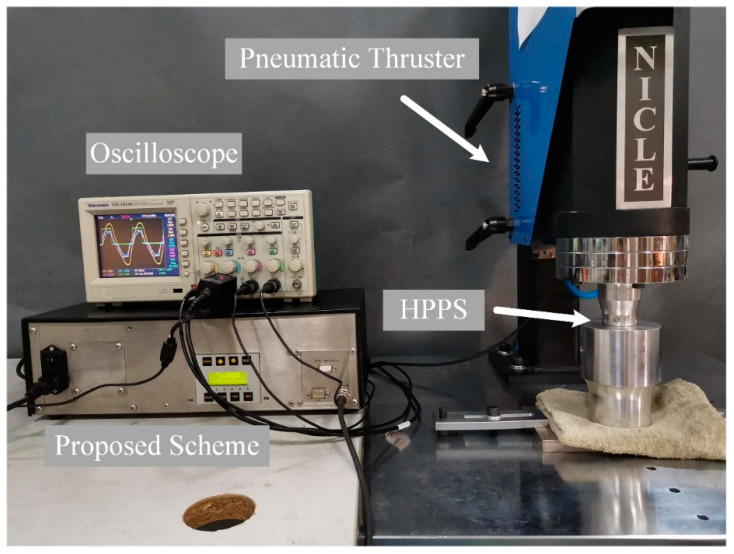
Experimental setup of frequency tracking verification.

**Figure 13:**
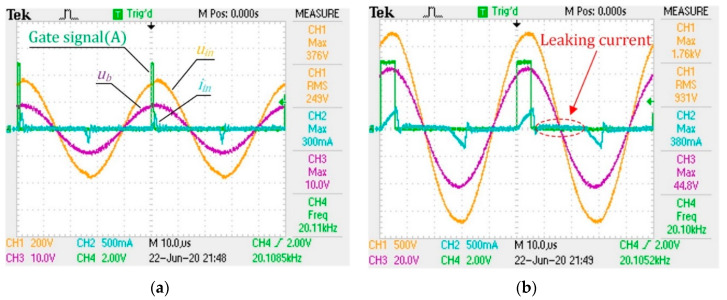

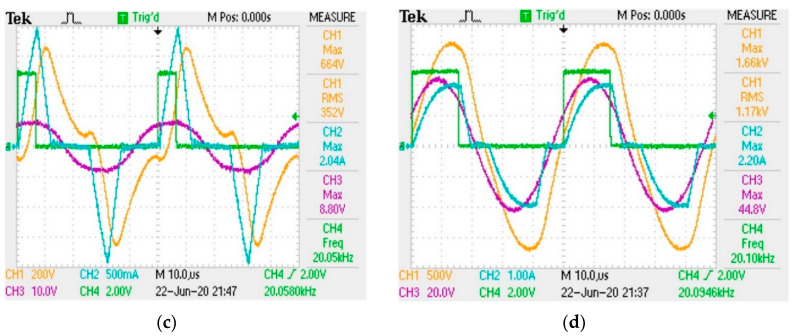
Waveforms of extreme operating conditions: (**a**) $R_{1}$ equals 7.40 Ω under 20% vibration amplitude; (**b**) $R_{1}$ equals 7.40 Ω under 100% vibration amplitude; (**c**) $R_{1}$ equals 500 Ω under 20% vibration amplitude; (**d**) $R_{1}$ equals 200 Ω under 100% vibration amplitude.

The experimental setup is demonstrated in Figure 12. Here, the HPPS is fixed on a pneumatic thruster and pressed against a damp cloth. We apply different loads to the HPPS by adjusting the cylinder pressure of the thruster. Meanwhile, the waveforms of $U_{in}$, $I_{in}$, $U_{b}$ and the gate signal A are measured by a Tektronix TDS 2024B oscilloscope, and the waveforms of four extreme operating conditions are shown in Figure 13. Here, 100% controlled vibration amplitude corresponds to about 1700 V of $U_{1}^{\prime }$. The sampling period of the oscilloscope is 0.04 μs in the experiments, and the accuracy is 0.04 per division for each channel. This result shows that the center of the pulse coincides with the peak of $u_{b}$, indicating that the frequency tracking meets the design. Meanwhile, the current waveforms are basically consistent with the analysis. The difference is caused by a weak leaking current in the high-resistance region, which may be attributed to the influence of the parasitic capacitance of the IGBT modules in the inverter.

**Figure 14:**
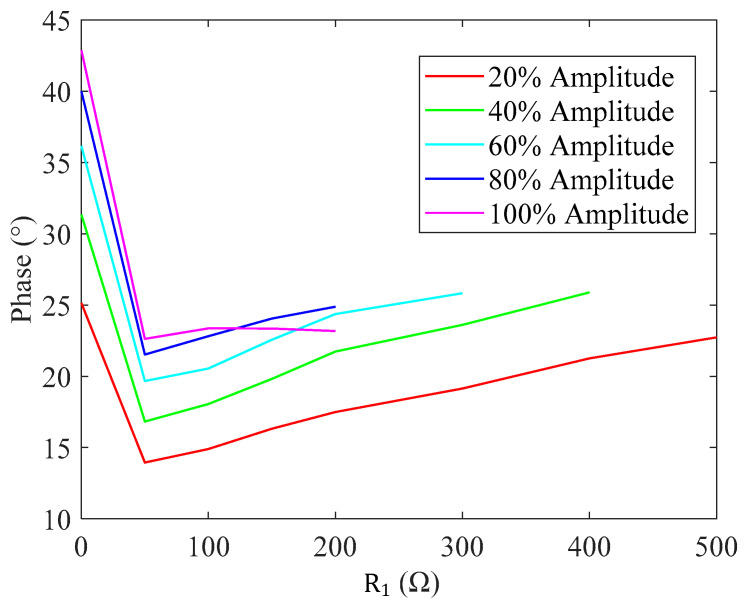
Phase deviation under different loads and vibration amplitudes.

Further, we perform Fourier transform to extract the fundamental wave of $I_{in}$, and the actual θ is calculated and demonstrated in Figure 14. This result shows that the phase difference is about 15°~25° under most operating conditions but relatively large under no load conditions ($R_{1}$ equals to 7.40 Ω). It is inferred that the load optimization frequency is slightly lower than $f_{p,}$ with θ near 20° in Section 2.2. The experiment result verifies this inference in which the actual excitation peak voltage is 1.76, 1.58, 1.58 and 1.62 kV when $R_{1}$ equals to 7.40, 50, 100 and 200 Ω, respectively.

### 5.2. Vibration Control Verification

**Figure 15:**
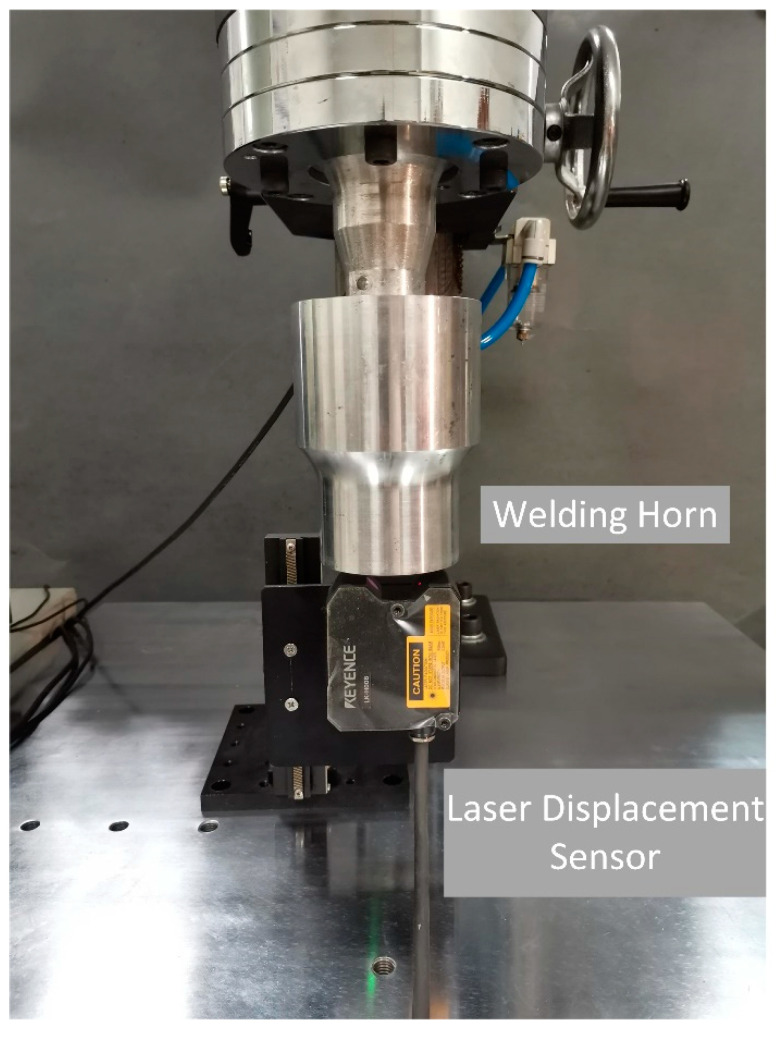
Experimental setup of vibration control verification.

**Figure 16:**
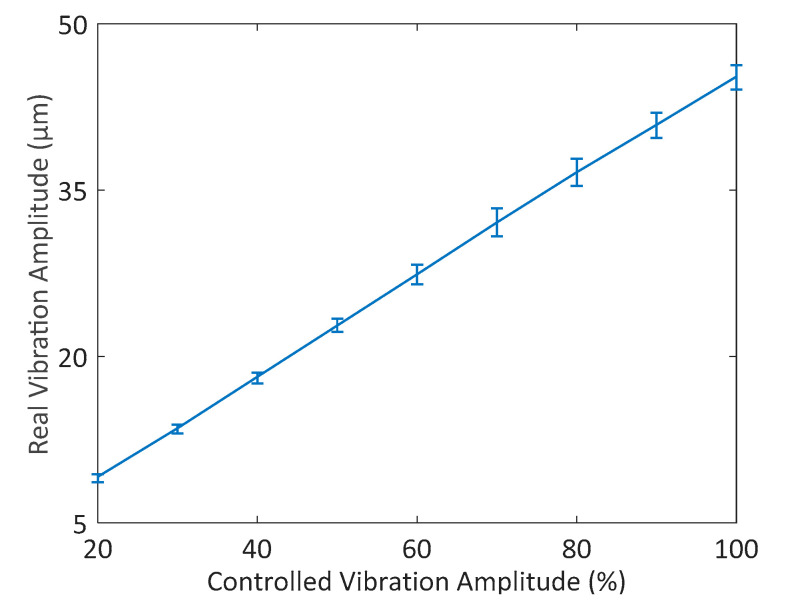
Real vibration amplitude under different control targets.

In this experiment, the vibration is tracked in different amplitudes by the proposed scheme and the actual vibration amplitude is measured by a KEYENCE LK-H008 laser displacement sensor. The measurement setup is shown in Figure 15. Each measurement is repeated five times, as shown in Figure 16. The result shows the linear relationship between our controlled vibration amplitude and the actual vibration amplitude. It verifies the reliability of our scheme in vibration control, which can be suitable for different processes.

### 5.3. Vibration Stability under Variable Load

In order to verify the stability of vibration control in varying load condition, we set target vibration amplitude to 30%, and increase the load gradually using water. Meanwhile, the laser senor measures the actual vibration amplitude. The proposed scheme also calculates and records $R_{1}$ in real time according to the equation:

(28) $$R_{1}=\frac{I_{T}\cos\theta }{U_{1}^{\prime }{\left(\omega C_{0}\right)}^{2}}.$$

**Figure 17:**
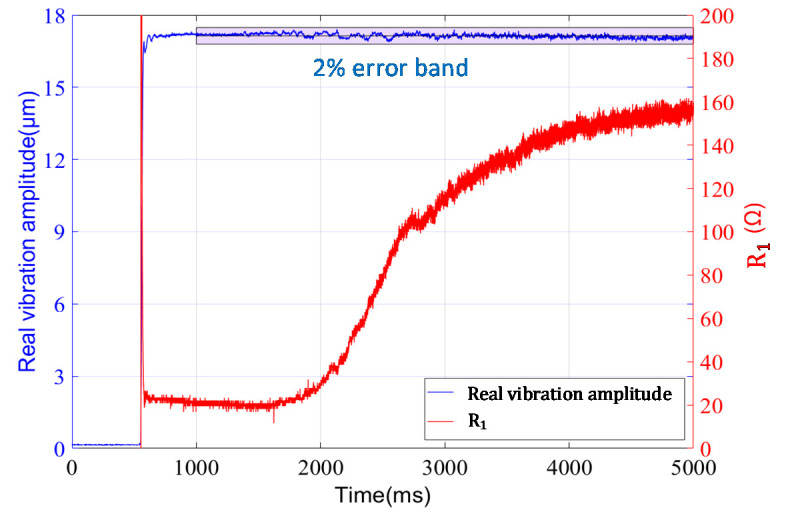
Variation in vibration amplitude under varying load.

The variations in the actual vibration amplitude and $R_{1}$ are shown in Figure 17. It shows that $R_{1}$ gradually increases 10 times after startup, while the fluctuation in the actual vibration amplitude is within 2%. This result verifies the feasibility in a wide range of loads. On the other hand, it also demonstrates the dynamic adaptability of our scheme. Although the capability of frequency tracking and vibration control is verified, the HPPSs under transient state have more complex characteristics, which should be further considered in the dynamic process.

## 6. Conclusions

This paper demonstrates that the proposed scheme is capable of frequency tracking and vibration control under a wide operating range. First, the impedance analysis indicates that the excitation current $I_{T}$ varies in a wide range under different loads near $f_{p}$. The analysis also indicates that a slight deviation in phase θ affects the impedance characteristics little. Especially, we suggest that the load optimization frequency with about 20° phase difference can help avoid the excessive rise up of the excitation voltage under high load conditions. Second, the electrical architecture is built, and the drive capability of the scheme is verified in wide range of loads and different vibration amplitudes. Then, the pulse-based phase detector (PBPD) is proposed, which can get the phase signal over a wide operating range with acceptable precision. The experiments verify the feasibility of PBPD and vibration amplitude control under the resistance range from 7.40 to 500 Ω and the vibration range from 20% to 100%. Finally, the experiment verifies that the accuracy of the vibration control is within 2% via a laser displacement sensor under varying load. The proposed scheme could be very useful for the HPPSs working in complex conditions, like ultrasonic welding and cutting.

## Figures and Tables

**Table 1:** Parameters of the BVD model for the HPPS.

Parameters	$R_{d}$ (Ω)	$C_{0}(\mathrm{nF})$	$C_{1}(\mathrm{nF})$	$L_{1}(\mathrm{mH})$	$R_{1}$ (Ω)
value	7.26	18.93	0.1696	370.4	7.40~500
